# Experiments on Calculi, in and out of the Body, Etc.

**Published:** 1868-11-15

**Authors:** T. Williams

**Affiliations:** Milwaukee, Wis.


### EXPERIMENTS ON CALCULI, IN AND OUT OF THE BODY, ETC.

BY T. WILLIAMS, M.D., MILWAUKEE, WIS.

*To the Editors* Chicago Medical Journal:

The following cases may be of some interest to your readers:

Case 1.—John Placer, æt. 37, sandy hair, and sanguine complexion, applied to me early in May, 1868, for treatment. Had been troubled with rheumatism since 1864, owing to exposure during the war; ankles and knee joints were considerably swollen, but not so much as to prevent his walking with a cane, without crutches. For the past six months had suffered a good deal with his back; pain, with a sense of fullness in the left kidney, especially before rising in the morning. For the past few days the pain had increased in the left kidney, and the sense of constriction and fullness was not relieved, as had previously been the case, on urinating. The pain was constant, and at times excruciating. He voided nearly the usual quantity of urine each day. The left testicle was painful and retracted, and there was a peculiarly unpleasant and annoying sensation in the base of the glans penis. On examination of the urine, it was found to be strongly acid. Renal calculi, supposed to be of uric acid, was diagnosed.

The bowels were opened freely with a saline draught; this was followed by thirty drops of deodorized fluid *Opium*, four times daily, and the patient was directed to take a tea-spoonfull of Coxe's "Hive Syrup" every two hours, preceded by a hot bath lasting one hour. The effect of this treatment was to give relief from the most urgent symptoms in about twenty-four hours. The testicle became pendant, but was preternaturally tender; the urine passed much more copiously than for several days, and the pain and fullness in the lumbar region subsided, owing probably to relaxation of the ureter at its exit from the pelvis of the gland, where the calculus was probably impacted. A mustard poultice was put over the kidney, until it blistered; the surface was dressed with savine ointment, which proved very irritating, and kept the spot quite sore.

The patient was kept on alkaline remedies, which kept the urine neutral or alkaline, until the first of September, three months; during this time his joints were rubbed with liniments, laved with alkaline solutions, blistered and poulticed persistently; the bowels were kept regular with alkaline laxatives, enemata, etc., and the state of the digestive organs improved with *Iron* and tonics. But on the first of September it was discouraging and vexatious to find the patient not one whit better than when he commenced. Frequently during this time the renal trouble had returned, and the same measures had to be resorted to repeatedly for its relief; the trouble in the back always returned. The result of this case, so far, had been quite similar to many others treated on the alkaline plan. I never could get the fine results from it that they do in the hospitals. I felt, in fact, disgusted with such poor results. If the theory is correct that rheumatism depends upon acidity of the circulation, it ought to be cured at all times, readily, by the administration of alkalies; and this is doubtless the proper mode of treatment in a certain type of cases in which some vice of the system causes the production of more uric acid than combines with the basic alkalines. The administration of lime, soda, etc., in these cases corrects the trouble for the time being, but does it remove the vice on which the disproportionate production of acid depends?

There seems to be another type of cases met with, which are due to a deficiency of acid, or an undue production of alkalies. It is certainly as preposterous to give alkalies in rheumatism and gout, with chalky or carbonate of lime deposits in the joints, as to administer alkalies for phosphate urinary deposits. In such cases, the alkaline element, not meeting sufficient acid to combine with, is left free, and is stranded in the joints, kidneys, liver, and heart, in greater or lesser quantities, giving rise to unendurable pains of a rheumatic or neuralgic chaaacter.

With these views, I commenced to give my patient *Phosphoric acid*, dilutum, twenty drops, three times a day, half hour before meals, in two ounces of *distilled* water. (This acid, from the extreme readiness with which it combines with lime, should never be administered in hard water.) For although his urine was naturally acid, it did not seem to exert any solvent power on the deposits of his system; and alkalies had proved equally useless. The patient's urine, on leaving off the alkalies and commencing the *Phosphoric acid*, became acid again within twenty-four hours; and from that time his improvement became marked and regular. About the first of October, he had several fits of the gravel, during which three small calculi, weighing respectively 5 grains, 3 grains, and 2 grains, passed down into the bladder and were voided with the urine, (suitable precautions having been taken to secure this result), after intense suffering. There was speedy and great relief experienced in the kidney immediately after the expulsion of the calculi. The patient's rheumatism had gradually improved, so that he felt better in his limbs than he had for near four years. The *Phosphoric acid* was continued, and sulphurated baths prescribed every other day. (At first 1½ ounces to the bath, gradually increased up to 3½.) The pain in the glans disappeared after the passage of the calculi. My theory is that the *Phosphoric acid* dissolved the little projections and virus of the calculi, allowing their expulsion.

I possessed no means of making a chemical analysis of these calculi, but they were very hard, uneven, and had the appearance of uric calculi. It occurred to me to make some experiments on the solubility. I placed the large one in dilute *Sulphuric acid*; the next largest in liquor *Potassa*, and the smallest in "Katalysine Water" from the Gettysburgh (Pa.) spring, claimed to possess a solvent power over calculi *in the body*. Twenty-four hours afterwards, the stones were just as when put in; the one in *Sulphuric acid* harder, if anything, than ever. I in turn placed them in a strong solution of *Iodide potassium*, *Sulphuric and Nitric ether*, alcohol, solutions of lime and of *Soda*, saliva, and gastric juice, (from a calf), without affecting their visible or sensible properties in the least. It is therefore fair to presume that none of these agents, internally administered, and necessarily largely diluted, would have affected them. I next tried the officinal *acidum phosphoricum dilutum*, which dissolved them all, with effervescence, in about twelve hours. The solution of the calculi in the acid gave it a peculiar but indescribable taste, reminding one of the odor of musk. The patient used nothing after September 1st but the acid and baths, and, when last seen, (November 1st, 1868,) was apparently over his rheumatism, though the joints remained a little stiff.

Case 2 will be more briefly referred to. The patient was a professional man, (a lawyer), whose business was purely intellectual. He had a well balanced and active brain; was a close student and an industrious scholar. The occasion of his consulting me was a fear which he entertained that he should fall a victim to softening of the brain, to which he thought he was exposed on account of the incessant activity of that organ. Mornings, he felt well, but when he went to his office and got to work hard, (writing briefs, etc.), the part of the head corresponding to the cerebrum would begin to feel *light;* a kind of dizzy, "foolish" feeling, free from ache or throbbing; and he would become quite nervous and tremulous. As the day wore on he could not work at all for this queer feeling in his head, and toward evening could not even read the daily paper without experiencing it. The slightest mental application seemed to affect him. Here was evidently a case in which the brain was denied a due proportion of some element necessary to its healthy action. The nervous mass, when employed in the complex operations concerned in the elimination of *thought*, consumes vast quantities of phosphorus. I suspected that this element was wanting. On further inquiry I learned that some alkaline mineral waters, which had been recommended to him as a tonic by a former medical adviser, perceptibly increased the trouble. The patient's urine was phosphatic. Here again the phosphoric acid of the system combining with the alkaline basis of the waters, still further diminished the supply the struggling brain so much needed. I directed these waters to be discontinued, and prescribed *Pyrophosphate of Iron* and dilute *Phosphoric acid*, with the effect of giving *immediate* relief. The patient prepared an able brief the same day he commenced this treatment without experiencing any of the head symptoms, but a resort to the mineral water, or the neglect of the acid, would any day insure their return. The phosphates disappeared from the urine, and it became acid within twelve hours.

In this age of great mental activity, there is an enormous expenditure of nervous force and vitality which induces physical debility and premature old age. No one cause, perhaps not all other caeses combined, contributes so much to abbreviating the space of human life. The average of life has steadily decreased from Noah down, and this decrease has kept pace with the advance of intellectual vigor and activity. I am much inclined to think that many modern physical organizations suffer materially for the lack of phosphorus, and that all of that article consumed in the manufacture of lucifer matches is needed by the brains of the present generation! Many weak, small framed and weak boned persons owe their physical degeneracy to the absence of a sufficient amount of phosphates during growth. The phosphates of iron, soda, potassa, and lime are more frequently indicated than is generally supposed. I have even thought that the growing tendency to indulge in beer and other malt liquors may arise from the deficiency of phosphorus, creating a kind of craving for a stimulant, not satisfied by cold water, and only temporarily by liquors, but seems to be satisfied by a draught of an acidulous nature. Many an overworked brain is given beer when it asks for phosphoric acid.

